# The Texas State Medical Association

**Published:** 1901-05

**Authors:** 


### TEXAS MEDICAL JOURNAL.

ESTABLISHED JULY, 1885.

PUBLISHED MONTHLY.—SUBSCRIPTION $1.00 A YEAR.

Vol. XVI. AUSTIN, MAY, 1901. No. 11.

### THE TEXAS STATE MEDICAL ASSOCIATION.

### Thirty-third Annual Meeting.

(*Abstract of Proceedings.*)

Galveston, Texas, April 23, 24, 25, 26, 1901.

The thirty-third annual meeting of the Texas State Medical Association was one of the largest, most interesting and most important gatherings of medical men that has assembled in some years, there being in attendance some two hundred representative physicians from all parts of the State. Eighty-one new members were admitted. There was much rejoicing and handshaking over the passage of the new medical practice bill, which goes into effect July 9th.

First Day.—Dr. H. P. Cooke, chairman of the committee of arrangements, called the body together. Rev. W. M. Harris, of the First Baptist Church, invoked divine blessing, following which Mr. James B. Stubbs, City Attorney, representing His Honor, Mayor Jones, delivered an address of welcome. Mr. Stubbs made a short talk, in which he tendered the visiting delegates the freedom of the city, and suggested that the convention each year hold its annual convention in Galveston, as the Texas State Bar Association does.

Mr. Stubbs was followed by Judge George E. Mann, representing the Chamber of Commerce, who, after extending the visitors welcome, also asked the association to consider Galveston as a permanent place of meeting each year, believing that this would do much to encourage the Texas State Medical College, which is located here.

On the part of the local physicians, Dr. George H. Lee delivered an address of welcome and concluded by asking the physicians during their visit here to investigate the sanitary conditions, stating in effect that Galveston had nothing to fear this summer in the way of epidemics as a result of unsanitary conditions. On behalf of the State Association Dr. B. E. Hadra, of Dallas, president of the Association, said that the royal welcome extended by the physicians, representative bodies and citizens of Galveston was not unexpected, as Galveston's hospitality was known far and wide.

SECRETARY'S REPORT: Dr. H. A. West, Galveston, the long-time secretary, reported on the state of the membership,—and on the deaths which had occurred since last meeting—as follows:

Deaths since last report:

Dr. A. E. McMahon, of Tolar, formerly of Marshall, an ex-member, died June 19, 1900.

Dr. A. H. Schenck, Kenney; died August 24, 1900.

Dr. A. V. Doak, Taylor, an ex-member; died September 14, 1900.

Dr. E. P. Becton, Austin, an ex-president; died January 14, 1900.

Dr. A. N. Denton, Austin; died March 14, 1901.

If other members or ex-members have died, the facts have not been brought to my notice.

THE TRANSACTIONS FOR 1900, a handsome volume—the best that has ever been issued, gotten out by the Von Boeckmann-Schutze Printing Company, of Austin, who for many years have secured the contract by competitive bid,—500 copies of same, 450 pages, handsomely illustrated,—cost, delivered to members, $540.56, or $1.09 per copy (including postage). [The details of the secretary's report of expenses, etc., are omitted.] There are twenty-three affiliating societies—all represented. Dr. West reports further as follows:

At the last meeting of the American Medical Association a committee was appointed as follows: Drs. J. N. McCormick, Bowling Green, Ky.; P. Maxwell Foshay, Cleveland, O.; George H. Simmons, Chicago, to consider and recommend a plan for a thorough organization of the medical profession of the country. At the present time there are about 1300 regular medical societies, most of which were organized and are acting independently of each other. This independency of organization and of action applies to State as well as other societies, each being based on a plan of its own, scarcely any two being organized alike. The result is a lack of uniformity or concert of action among these bodies.

1. The first object of the committee is, therefore, to suggest a remedy whereby the State bodies may be brought together.

2. In most of the States no systematic attempt is made to organize local societies on a specific plan. Consequently there can be no united effort made, political or otherwise. A remedy must be considered that will change this condition.

3. The committee believes that if the profession is to become a united body, the foundation must rest in the county society; these to be branches of the State society, all organized on a specific plan as made by the State society. Likewise all State societies must be organized on a specific plan as branches of the American Association, and as agreed upon by all these State societies.

Conforming to the request of the committee I sent a blank to the treasurer of each society on our list, and obtained replies to 15 of the number. A synopsis of the information thus obtained was sent to Dr. Simmons; as the committee had not yet formulated its report, I am unable to present it for your consideration. In this connection I beg leave to call your attention to the amendment to the by-laws reciting the basis for affiliation.

In page 40, transcript, which now comes up for settlement—also the amendment increasing the salary of the treasurer to $150. I am requested by Dr. J. H. Grant, of Palestine, to ask your co-operation with a committee of the State Dental Association, of which he is chairman, on text-books for our schools. We are requested by the committee of arrangements of the New York State Medical Association to send delegates to the meeting of that body, which is to be held in New York City, October 21-24, 1901. The possibility of the American Association coming South at its meeting in 1902, has suggested to me the propriety of extending an invitation to that body to meet in Texas. If it meets with your approval it would be well for us to unite upon the most eligible point and send a strong delegation to St. Paul to urge our claims.

I regret to say that the old volumes of transactions were very much injured by the flooding of my office by the storm of September 8.

I beg to advise you that in accordance with resolutions adopted last year at Waco that the Surgeon General of the M. H. S., has detailed Past Assistant Surgeon C. P. Wertenbaker, of New Orleans, to represent the service at this meeting. [Dr. Wertenbaker was present, and made a neat speech. He met with a cordial reception and made many friends, both for himself and the service he represented.]

THE TREASURER'S REPORT showed a balance on hand, after all expenses were paid, of $1316. About $1500 more was paid in at the meeting, so that the Association is in solid financial condition.

At the afternoon session the program was resumed, the section on Practice of Medicine being called. Dr. J. D. Law, of Belton, chairman, delivered the address.

Owing to the absence of Dr. J. W. Embree, of Belton, and Dr. L. Ashton, of Dallas, papers prepared by them were not read. Dr. Embree was to have read a paper entitled "Is Fever a Conservative or Non-Conservative Process?" and the subject of Dr. Ashton's paper is "Acute Interstitial Nephritis, Pathology and Treatment." Papers entitled "Further Observations on the Medical Aspects of the Galveston Storm," by Dr. H. A. West, of Galveston [Dr. West's paper will appear in this journal next issue.—Ed.]; Serum Therapy, Its Uses and Limitations," by Dr. J. P. McElvey, of Temple, "Twentieth Century Smallpox," by Dr. J. T. Benbrook, of Rockwall; "The Epidemic of Cerebro-Spinal Meningitis in Texas in 1898 and 1899," by I. J. Jones, of Austin; a paper by Dr. W. R. Blailock, of McGregor, and "Malarial Infection, Its Prevention," by Dr. Walter Shropshire, were read and discussed.

BETTER THAN TRADING STAMPS OR A CHROMO: Just before the afternoon session adjourned, Secretary West stated that a local druggist desired it announced that he would give to every member of the Association one share of stock in an oil company, and to the president fifty shares. The announcement was greeted with applause. The convention then adjourned until 8 p. m.

EVENING SESSION. The evening session was confined to section on Obstetrics and Diseases of Children. Dr. W. Keiller, of Galveston, delivered an address on the subject of "The Axis Traction Forceps." Papers were also read by Dr. Malone Duggan, of Eagle Pass, on "Puerperal Infection—Inquiry as to Sources and the Physician's Responsibility." Dr. T. W. Shearer, of Wallisville, a paper on "Concealed Antepartum Hemorrhage," and Dr. J. F. Y. Paine reported on the obstetrical service of the John Sealy hospital for the ten years ending with 1900.

SECOND DAY.—MORNING SESSION. The session was devoted to executive business.

Dr. J. T. Wilson, of Sherman, chairman of the committee on medical legislation, presented his report, citing the passage of the bill by the last Legislature regulating the practice of medicine in this State.

Dr. Norsworthy moved that a committee of one from each congressional district of the State be appointed by the president of the Association to select the names of members for appointment on the State board of medical examiners under the new law.

Dr. H. A. West, of Galveston, as a substitute, moved that the special committee be appointed by the members of the Association, one from each congressional district of the State.

The substitute carried and the following committee was appointed: First, Dr. R. T. Morris, Houston; Second, Dr. W. B. Collins, Lovelady; Third, Dr. Joe Becton, Greenville; Fourth, Dr. J. Y. Bradfield, Daingerfield; Fifth, Dr. J. E. Gilcreest, Gainesville; Sixth, Dr. W. A. Watkins, Kemp; Seventh, Dr. M. L. Graves; Eighth, Dr. A. J. Grey, Comanche; Ninth, Dr. E. M. Thomas, Georgetown; Tenth, Dr. H. A. West, Galveston; Eleventh, Dr. H. W. Crouse, Victoria; Twelfth, Dr. R. E. Moss, San Antonio, and Thirteenth, Dr. J. E. Dodson, Vernon.

The committee presented the following names:

First District, Dr. J. W. Scott, Houston.

Second, Dr. J. H. Evans, Palestine.

Third, Dr. T. J. Bell, Tyler.

Fourth, Dr. D. J. Jenkins, Daingerfield.

Fifth, Dr. J. T. Wilson, Sherman.

Sixth, Dr. J. C. Loggins, Ennis.

Seventh, Dr. Taylor Hudson, Belton.

Eighth, Dr. R. H. Rush, De Leon.

Ninth, Dr. M. M. Smith, Austin.

Tenth, Dr. John C. Jones, Gonzales.

Eleventh, Dr. J. H. Reuss, Cuero.

Twelfth, Dr. Frank Paschal, San Antonio.

Thirteenth, Dr. P. C. Coleman.

From the Satte at large:

Dr. Walter Shropshire, Yoakum.

Dr. Sam R. Burroughs, Buffalo.

Dr. W. R. Blailock, McGregor.

Dr. A. C. Scott, Temple.

Dr. A. B. Gardner, Bellville.

The report of the committee was adopted.

The Afternoon Session was occupied by the surgical section. Dr. I. N. Suttle, of Corsicana, chairman, delivered the address in surgery. The following papers were read:

Dr. J. E. Thompson, of Galveston, on "The Treatment of Hare Lip," and Dr. H. A. Barr, of Beaumont, on "Surgery of the Cranium and Brain--Report of Three Cases," Dr. S. T. Turner, of El Paso on "Dry or Wet Dressing in Minor Surgery," Dr. William Keiller, of Galveston, on "The Surgical Anatomy of the Ischio-Rectal Region and Its Bearing on Piles and Fistula in Ano, With a New Operation for the Former."

SECTION ON MEDICAL JURISPRUDENCE. Dr. J. S. Lankford, San Antonio, chairman, delivered the address. The following papers were read: "Jury Trials of the Insane," by Dr. Marvin L. Graves, of San Antonio; "An Ideal Eleemosynary System," by Dr. John E. Turner, of Terrell.

After a discussion of the various papers the meeting adjourned until 8 o'clock in the evening.

At the evening session papers under the head of medical jurisprudence were read by Dr. R. H. Harrison, of Columbus, on "Medical Jurisprudence as a Branch of State Medicine," and by Dr. J. T. Wilson, of Sherman, on "The Importance and Difficulties of Insanity in Its Medico-Legal Aspect."

Memorial services were then held and suitable resolutions respecting the several deceased members were adopted. Dr. J. W. McLaughlin paid a beautiful and touching tribute to the memory of Dr. Becton.

THIRD DAY.—MORNING SESSION. Executive session, after which section work was resumed, and the program was carried out, the section on ophthalmology being called. This section had not finished its work when the Association took a recess from 2 to 4 and the delegates and their ladies were taken on a steamboat excursion on the bay—along the entire water front, and as far down as the jetties. It was a thoughtful attention on the part of Chairman Cooke and his associates on the arrangement committee, and much appreciated. On the excursion--another thoughtful attention—the party was regaled with Galveston beer and sandwiches, to which the sharp appetites engendered by the cool sea breeze did entire justice.

At the morning session a resolution was adopted that when the Association procures its new charter the trustees, Drs. B. E. Hadra, Taylor Hudson, R. F. Miller, H. P. Cooke and H. A. West, be instructed to ratify the present constitution and by-laws and officers elected at this meeting.

The resignation of Drs. Thomas G. Duncan, of Victoria, and F. G. Eidman, of Houston, were accepted.

A committee will be appointed to confer with Dr. J. H. Grant of the State Dental Association, on the selection of text-books for public schools.

The American Medical Association will be invited to hold its convention in 1902 in Texas. The president and one delegate from the Texas Association will attend the national convention.

The discussion of the methods for the prevention of smallpox, which was postponed Wednesday evening, was next taken up, and the matter consumed most of the morning.

Dr. I. J. Jones, of Austin, and Dr. Turner, of Terrell, spoke on the subject.

The report of Dr. R. H. Harrison, chairman of the Board of Health, was received and adopted, as was also the report of Dr. J. B. Massie, chairman of the committee on medical legislation.

Election of Officers. The report of the nominating committee was received and adopted unanimously. The committee named two members for the office of the president of the Association, but friends of Dr. T. J. Bell, of Tyler, acting under instructions from Dr. Bell, withdrew his name, and Dr. Taylor Hudson, of Belton, was elected by acclamation, president of the Texas Association.

Officers for the ensuing year; report of nominating committee.

President, Dr. Taylor Hudson, Belton, Texas.

1st Vice-President, Dr. S. C. Red, Houston, Texas.

2nd Vice-President, Dr. J. W. Nixon, Gonzales, Texas.

3rd Vice-President, Dr. W. A. Watkins, Kemp, Texas.

Members Judicial Council: Dr. T. J. Pier, Carmine, Texas; Dr. W. C. Blailock, Kosse, Texas; Dr. J. E. Thompson, Galveston, Texas; Dr. S. T. Turner, El Paso, Texas.

Orator: Dr. W. H. Moore, Runge, Texas.

Publishing Committee: Dr. H. A. West, Sec., Chairman; Drs. H. P. Cooke and I. M. Cline, Galveston.

Time and place of meeting in 1902, last Tuesday in April, El Paso, Texas. Dr. S. T. Turner, El Paso, Chairman Committee on Arrangements.

Committee on Medical Legislation (continued as last year).

Committee on County Organization, continued, with the addition of Dr. J. T. Carter, of Fayette county, vice Schenck, deceased.

We also recommend that the American Medical Association be invited to hold its annual meeting, 1902, in Houston, Texas.

Respectfully submitted,

S. C. Coleman,

President Nominating Committee.

J. C. Loggins, Secretary.

RECEPTION AT HARMONY HALL.

Rosenberg Hall was filled with members of the Texas State Medical Association, their ladies and friends last night, to hear the annual address, delivered by Dr. B. E. Hadra, of Dallas, the retiring president of the Association. Dr. John O. McReynolds, of Dallas, who was to deliver the annual oration, was detained in Dallas, and this feature of the evening program had to be omitted.

From Rosenberg Hall the members of the Association and their invited guests repaired to Harmony Club to attend the reception and banquet given in honor of the visiting physicians by the citizens of Galveston. It was a delightful affair, devoid of the conventional toast making and formalities of such occasions. The dining hall was tastefully decorated and the banquet table was laden with a tempting array of edibles and delicacies. An orchestra dispensed sweet music during the progress of the feast, and at its conclusion dancing was indulged in until a late hour.

FOURTH DAY.—The following resolution, offered by Dr. R. H. Harrison, of Columbus, was unanimously adopted:

"Whereas, the report has gone abroad that the sanitary condition of Galveston is in a very unsatisfactory condition; therefore be it

Resolved, that the Texas State Medical Association congratulates the people of Galveston upon the excellent sanitary condition in which they have succeeded in placing their city so soon after the dire disaster to which they were so lately subjected.

To anyone familiar with the deplorable condition existing here a few months ago, the achievement is almost incredible. While Galveston has heretofore been noted for its excellent sanitary condition, there is no time in its previous history where it has presented a more favorable aspect than now.

The attendance upon the sessions was larger this year than at any time within the past ten years, the daily attendance averaging 200 representative physicians from various parts of the State. During the meeting 81 new members were admitted and the papers read and subjects discussed were entertaining and instructive.

The section on pathology was taken up, and the chairman, Dr. Allen J. Smith, of Galveston, delivered an able address. Then followed a paper by Dr. M. Charlotte Schaefer on the subject of a new form of intestinal parasite in Texas. Dr. J. T. Moore read a paper on malarial nephritis. A very remarkable case of appendicitis, where the vermiform appendix passed through the intestines, was the subject of a paper read by Dr. J. M. Frazer, of Belton, with the specimen exhibited.

The subject of the admission of the insane to asylums was taken up and discussed, and the following committee appointed to endeavor to bring about proper legislation on the subject: Dr. William Keiller, of Galveston; Dr. M. L. Graves, of San Antonio; Dr. John S. Turner, of Terrell; Dr. I. E. Clark, of Schulenburg, and Dr. A. B. Gardner, of Bellville.

The following committee on transportation to secure rates for the El Paso meeting of the Association in 1902 was appointed: Dr. J. R. Stewart, of Houston; Dr. A. C. Scott, of Temple; Dr. B. F. Eads, of Marshall, Dr. Amos Graves, of San Antonio; Dr. Bacon Saunders, of Fort Worth, and Dr. S. T. Turner, of El Paso, chairman of the committee.

A committee consisting of Drs. J. F. Y. Paine, Allen J. Smith and W. S. Carter, of Galveston, and Walter Shropshire, of Yoakum, was appointed to draft amendments to the constitution and by-laws relative to the rearrangement of sections and executive business. The committee is to make its report at the next meeting of the Association.

A resolution was adopted memorializing the Board of Regents to establish a chair of State Medicine and Public Hygiene at the State Medical College.

The following resolution of thanks was unanimously adopted and the meeting brought to a close:

At the close of this, one of the largest meetings of the Texas State Medical Association, be it

Resolved, that we hereby tender our thanks to the medical profession of Galveston for their very hospitable and complete entertainment of this Association; to the ladies of the city for their graceful presence and untiring efforts, which added so much to the enjoyment of the meeting; to the *Galveston News*, the *Galveston Tribune* and the *Houston Post* for their complete reports of the meetings; to the Tremont and other hotels for their courtesies.

